# Variation in pigmentation gene expression is associated with distinct aposematic color morphs in the poison frog *Dendrobates auratus*

**DOI:** 10.1186/s12862-019-1410-7

**Published:** 2019-04-18

**Authors:** Adam M. M. Stuckert, Emily Moore, Kaitlin P. Coyle, Ian Davison, Matthew D. MacManes, Reade Roberts, Kyle Summers

**Affiliations:** 1 Department of Biology, East Carolina University, Greenville, North Carolina USA; 2 Hubbard Center for Genome Studies, University of New Hampshire, Durham, New Hampshire USA; 3 Department of Molecular, Cellular & Biomedical Sciences, University of New Hampshire, Durham, New Hampshire USA; 4 Department of Biological Sciences, North Carolina State University, Raleigh, North Carolina USA

**Keywords:** Aposematism, Coloration, Color variation, Poison frog

## Abstract

**Background:**

Color and pattern phenotypes have clear implications for survival and reproduction in many species. However, the mechanisms that produce this coloration are still poorly characterized, especially at the genomic level. Here we have taken a transcriptomics-based approach to elucidate the underlying genetic mechanisms affecting color and pattern in a highly polytypic poison frog. We sequenced RNA from the skin from four different color morphs during the final stage of metamorphosis and assembled a de novo transcriptome. We then investigated differential gene expression, with an emphasis on examining candidate color genes from other taxa.

**Results:**

Overall, we found differential expression of a suite of genes that control melanogenesis, melanocyte differentiation, and melanocyte proliferation (e.g., *tyrp1, lef1, leo1,* and *mitf*) as well as several differentially expressed genes involved in purine synthesis and iridophore development (e.g., *arfgap1, arfgap2, airc,* and *gart*).

**Conclusions:**

Our results provide evidence that several gene networks known to affect color and pattern in vertebrates play a role in color and pattern variation in this species of poison frog.

**Electronic supplementary material:**

The online version of this article (10.1186/s12862-019-1410-7) contains supplementary material, which is available to authorized users.

## Background

Color and pattern phenotypes have long been of interest to both naturalists and evolutionary biologists [1, 2]. Part of this interest derives from the association of this phenome with selective pressures such as mate choice [3] and predation [4]. Species with morphological phenotypes directly tied to survival and reproduction provide excellent opportunities to study the genetic underpinnings of color and pattern, precisely because these phenotypes are so obviously linked to survival.

Aposematic species rely on color and pattern to warn predators, but in many cases these color and pattern phenotypes are extremely variable, often changing over short geographic distances or even exhibiting polymorphism within populations [5, 6]. Theory has long predicted that aposematic species should be monomorphic because predators learn a common signal, and thus aposematic individuals with a different phenotype should be selected against [2, 7]. While predator variation and drift alone may be sufficient to create phenotypic variation, a variety of alternative selective pressures can act on the aposematic signal to produce and maintain this variety (reviewed in [8]).

Research on the production of color and pattern early in life in polytypic species (those that vary in discrete phenotypes over geographical space) has been limited, especially in vertebrates. Differences in color and pattern in some highly variable aposematic species seem to be determined by a small number of loci [9–12]. However, the majority of the research on the underlying genetic architecture associated with varied color and patterns in aposematic species has been done in the Neotropical butterflies of the genus *Heliconius.* While this work has been highly informative, it remains unclear whether these trends are generally applicable to other systems, including in vertebrates.

Many of the Neotropical poison frogs (family Dendrobatidae) exhibit substantial polytypism throughout their range [6, 13]. Despite being one of the better characterized groups of aposematic species, our knowledge of the mechanisms of color production in this family is quite limited. In addition, there is little information on the genetics of color pattern in amphibians generally. While modern genomic approaches, especially high-throughput sequencing, have recently provided extensive insights into the genes underlying color pattern variation in fish [14, 15], reptiles [16], birds [17] and mammals [18–20], there have been few genomic studies of the genetic basis of color patterns in amphibians. This is in part because amphibian genomes are often large and repetitive. For example the strawberry poison frog (*Oophaga pumilio*) has a large genome (6.7 Gb) which is over two-thirds repeat elements [21]. The dearth of amphibian data is an important gap in our knowledge of the genomics of color and pattern evolution, and the genetic and biochemical pathways underlying color pattern variation across vertebrates.

Amphibians exhibit extremely varied colors and patterns, and these are linked to the three structural chromatophore types (melanophores, iridophores, and xanthophores) and the pigments and structural elements found within them (e.g. melanins, guanine platelets, and pteridines; Mills & Patterson 2009). Melanophores and the melanin pigments they contain are responsible for producing dark coloration, particularly browns and blacks, and are also critical to the production of darker green coloration [22]. Blue and green coloration in amphibians is generally produced by reflectance from structural elements in iridophores [23]. Iridophores contain guanine crystals arranged into platelets that reflect particular wavelengths of light, depending on platelet size, shape, orientation and distribution [16, 23, 24]. Generally speaking, thicker and more dispersed platelets reflect longer wavelengths of light [16]. Combinations of iridophores and xanthophores or erythropores containing carotenoids or pteridines (respectively) can produce a wide diversity of colors [16]. Xanthophores are thought to be largely responsible for the production of yellows, oranges, and reds in amphibians. The precise coloration exhibited is linked to the presence of various pigments such as pteridines and carotenoids that absorb different wavelengths of light [22].

**Fig. 1:**
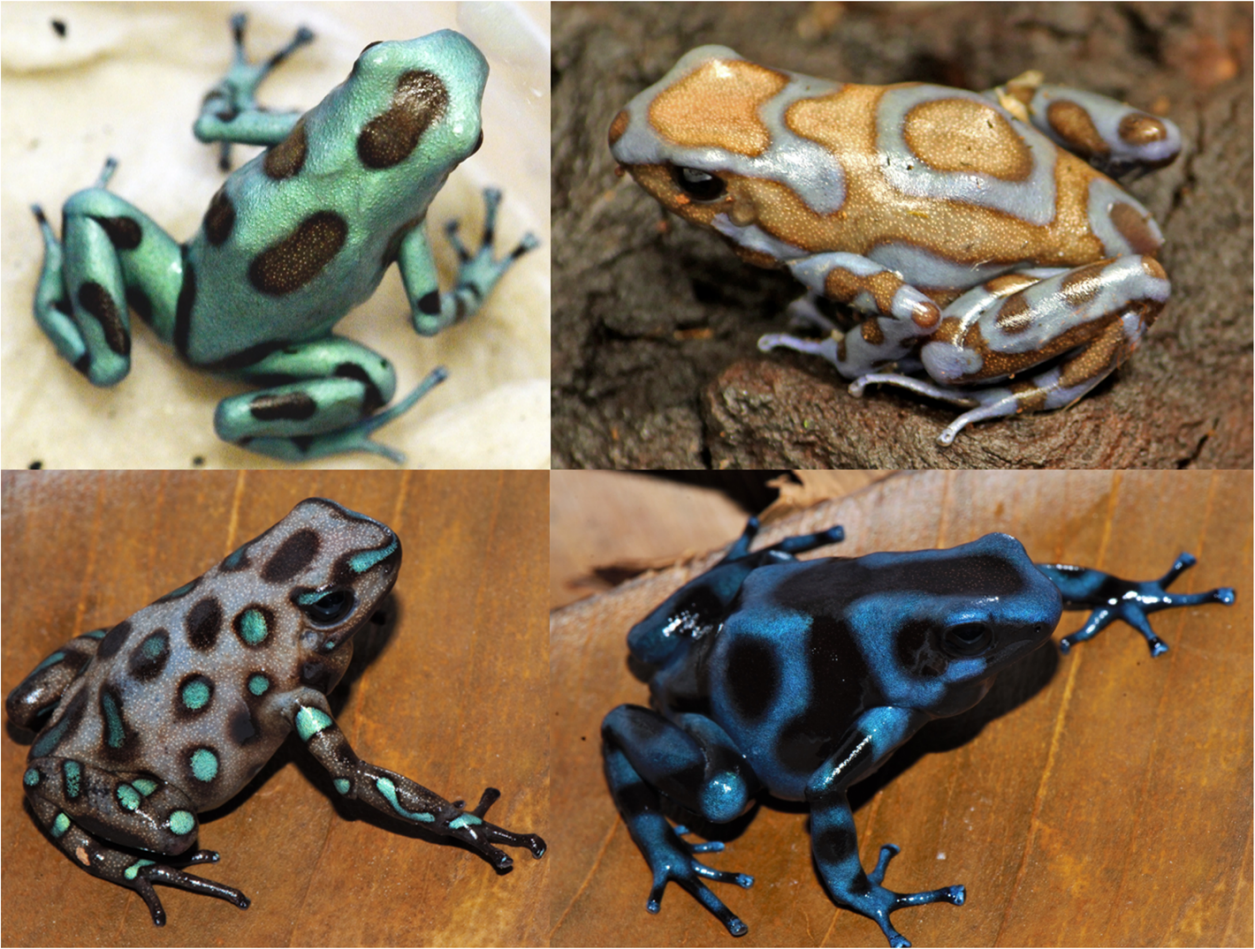
Normative depictions of the four captive morphs used in this study. Color morphs clockwise from top left: microspot, super blue, blue and black, San Felix. Microspot and super blue photographs courtesy of ID, blue-black and San Felix photos were provided by Mark Pepper at Understory Enterprises, LLC. Pictures used with permission

In order to better understand the genetic mechanisms affecting the development of color and pattern, we examined four different captive bred color morphs of the green-and-black poison frog (*Dendrobates auratus*). The San Felix and super blue morphs both have a brown dorsum, with the former having green spotting, and the latter typically having light blue markings (often circular in shape), sporadically distributed across the dorsum. The microspot morph has a greenish-blue dorsum with small brownish-black splotches across the dorsum. Finally, the blue-black morph has a dark black dorsum with blue markings scattered across the dorsum that are typically long and almost linear. Photographs of frogs from these morphs in captivity are found in Fig. 1. We used an RNA sequencing approach to examine gene expression and characterize the skin transcriptome of this species. In addition to assembling a de novo skin transcriptome of a species from a group with few genomic resources, we compared differential gene expression between color morphs. We focused on differential gene expression in a set of a priori candidate genes that are known to affect color and pattern in a variety of different taxa. Finally, we examined gene ontology and gene overrepresentation of our dataset. These data will provide useful genomic and candidate gene resources to the community, as well as a starting point for other genomic studies in both amphibians and other aposematic species.

## Results

### Transcriptome assembly

We used the Oyster River Protocol [25] to assemble a transcriptome; this protocol uses a series of different transcriptome assemblers and kmer lengths, ultimately merging them into a single transcriptome. After conducting the Oyster River Protocol for one random individual per color morph and merging them together, we were left with a large transcriptome containing 597,697 transcripts. We examined the BUSCO and transrate scores for each morph’s transcriptome, as well as for the transcriptome created by orthomerging these four assemblies (Table 1). BUSCO and transrate scores were computed using the full, cleaned read dataset from all samples. Given the poor transrate score of our final, merged assembly we selected and used the good contigs from transrate (i.e., those that are accurate, complete, and non-redundant), which had a minimal effect on our overall BUSCO score. In total, our assembly from the good contigs represents 160,613 individual transcripts (the “full assembly” in Table 1). Overall, our annotation to the combined *Xenopus, Nanorana, Rana,* and UniRef90 peptide databases yielded 76,432 annotated transcripts (47.5% of our transcriptome).

**Table 1:** Assembly metrics for each of our assembled transcriptomes. Metrics for the full assembly were calculated using the full, cleaned dataset. BUSCO scores represent the percentage of completion (i.e., 100% is an entirely complete transcriptome)

	Transrate score	Transrate optimal score	BUSCO score
Blue-black	0.05446	0.40487	96.3%
Microspot	0.04833	0.35907	94.0%
San Felix	0.0556	0.35718	88.1%
Super blue	0.0521	0.38094	96.0%
Full assembly	0.01701	0.13712	95.8%

### Differential expression and fixed variants

**Fig. 2:**
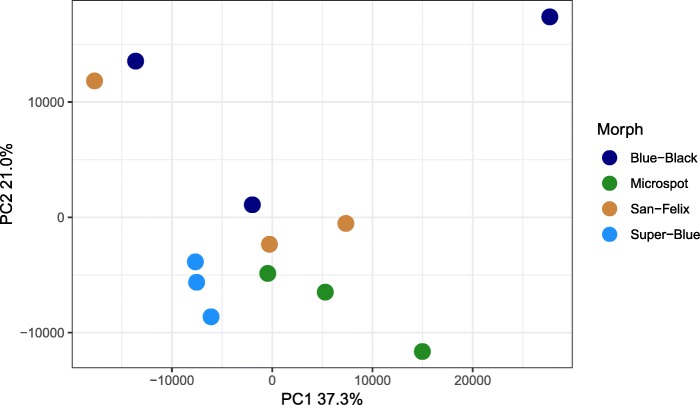
Principal component analysis indicating general within-morph similarity in transcript abundance within our dataset. PCA computation was normalized as transcripts per million. Each dot indicates one individual and the percentage of variation explained by the axes are presented

Our results indicate that there are distinct differences in expression between color morphs (Fig. 2). Principal component 1 explained 37.3% of the variation and principal component 2 explained 21.0% of the variation. We successfully mapped 81.6% ± 1.6% of our reads to our reference transcriptome. When we tested for differential expression, we found a total of 2845 differentially expressed transcripts among color morphs (1.77% of our transcriptome; Additional file 1: Table S1). We identified a total of 2172 SNPs on 1151 contigs. Of these, we found 28 SNPs on a contig with an annotated color gene and also alternately fixed among color morphs; these represent 16 unique candidate color genes (Additional file 2: Table S2).

### Pathway analyses

**Fig. 3:**
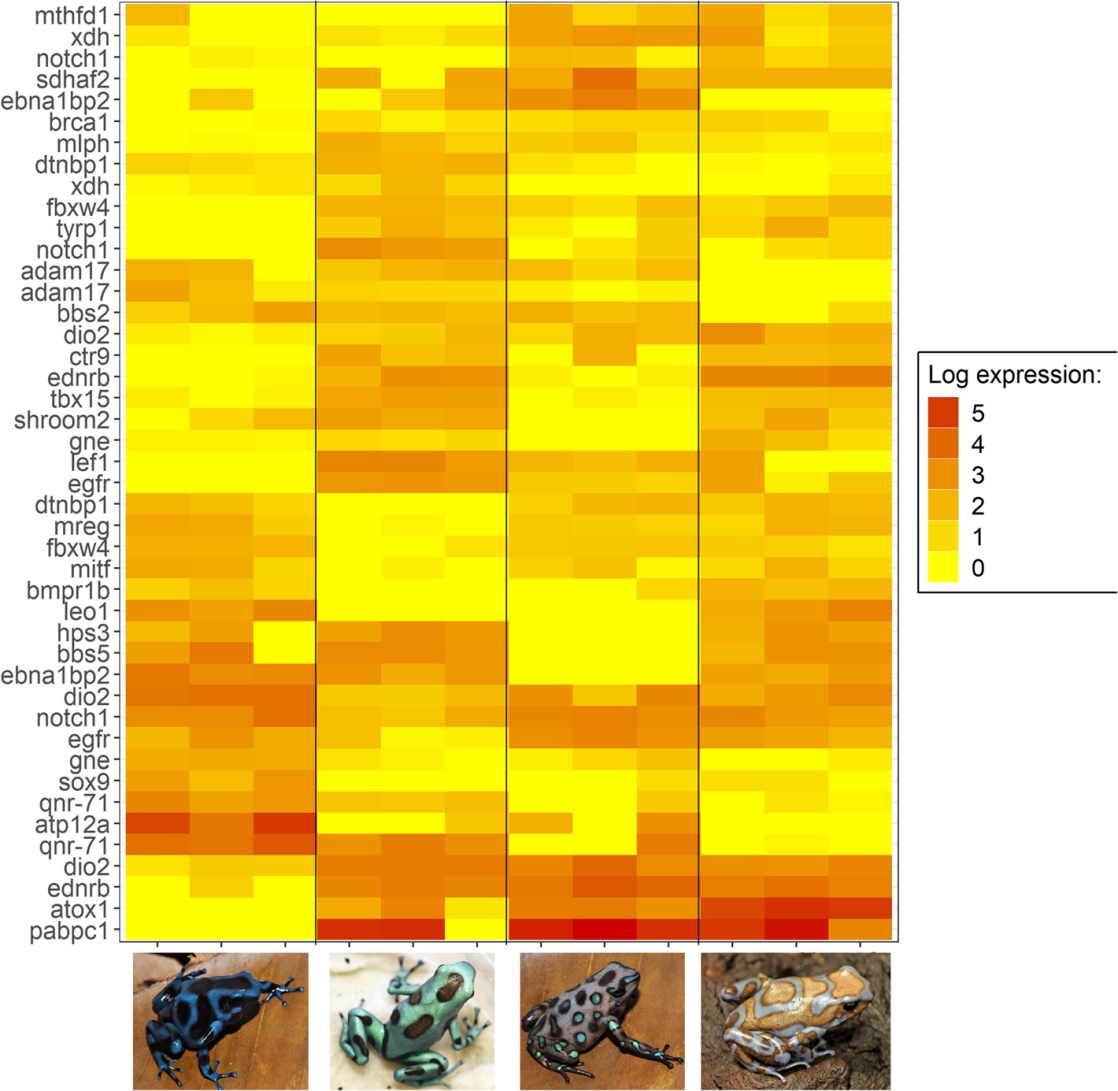
Log-fold expression (transcripts per million) levels of putatively melanin related genes that are differentially expressed between color morphs in *Dendrobates auratus.* Each individual is represented on the x-axis, and the y-axis represents expression levels for each transcript that annotated to an melanophore-related gene. Genes represented more than once mapped to multiple transcripts. Expression for this heatmap was calculated using transcripts per million in Kallisto, to which we added 1 and log transformed the data (i.e., expression = log(transcripts per million + 1). Microspot and super blue photographs courtesy of ID, blue-black and San Felix photos were provided by Mark Pepper at Understory Enterprises, LLC. Pictures used with permission

**Fig. 4:**
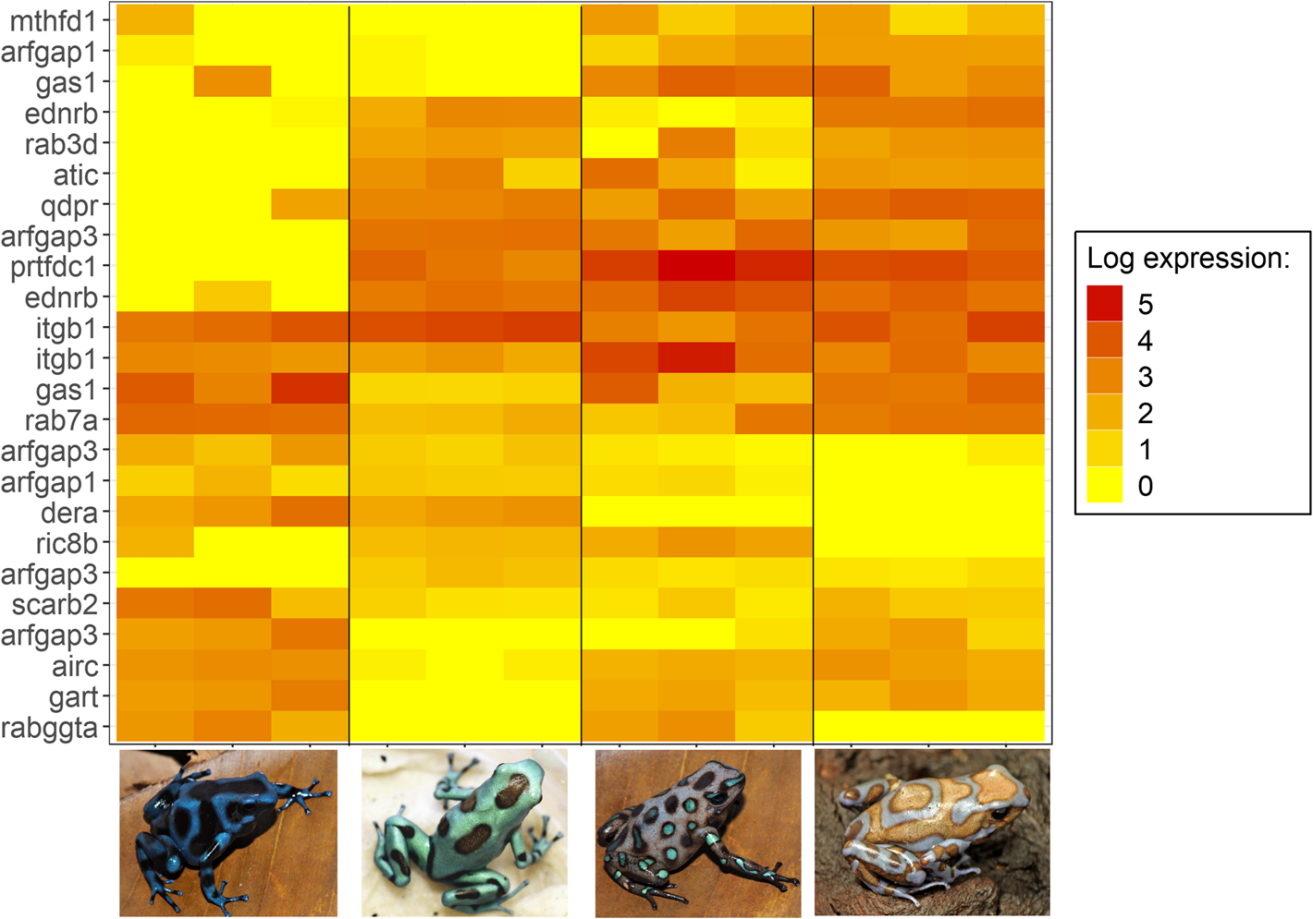
Log-fold expression (transcripts per million) levels of putatively iridophore-related genes that are differentially expressed between color morphs in *Dendrobates auratus.* Each individual is represented on the x-axis, and the y-axis represents expression levels for each transcript that annotated to an iridophore-related gene. Genes represented more than once mapped to multiple transcripts. Expression for this heatmap was calculated using transcripts per million from Kallisto, to which we added 1 and log transformed the data (i.e., expression = log(transcripts per million + 1)). Microspot and super blue photographs courtesy of ID, blue-black and San Felix photos were provided by Mark Pepper at Understory Enterprises, LLC. Pictures used with permission

**Fig. 5:**
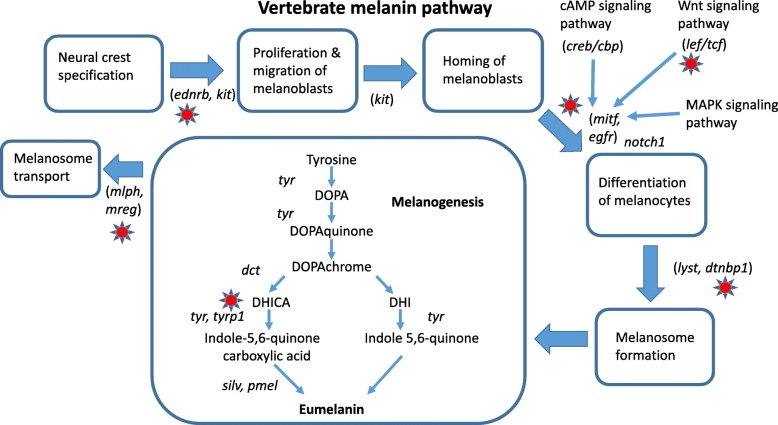
Melanin pigmentation pathway in vertebrates. Here we highlight differentially expressed genes in our dataset with a red sun

**Fig. 6:**
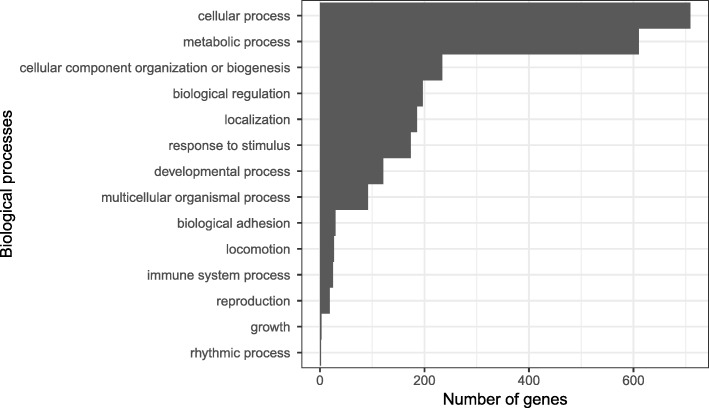
Gene ontology terms from PANTHER. Bars depict the number of differentially expressed genes in each biological process GO category

**Fig. 7:**
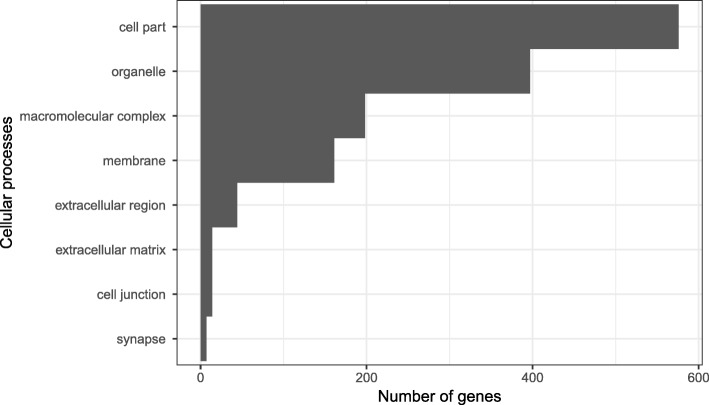
Gene ontology terms from PANTHER. Bars depict the number of differentially expressed genes in each cellular process GO category

**Fig. 8:**
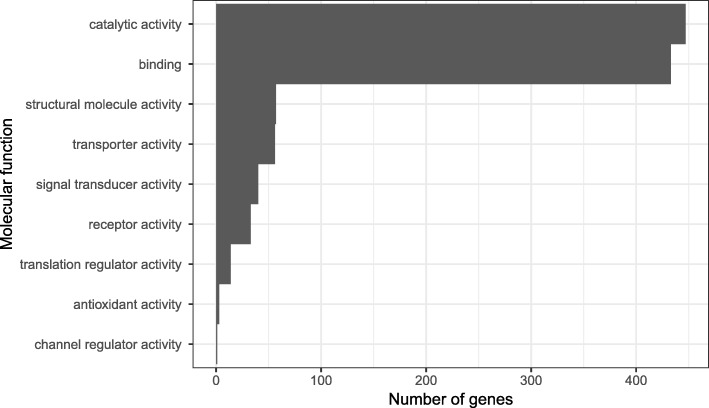
Gene ontology terms from PANTHER. Bars depict the number of differentially expressed genes in each molecular function GO category

From our list of candidate color genes, we found 58 differentially expressed transcripts (q value < 0.05) associated with 41 candidate color genes in total (see Table 2 and Figs. 3 and 4). Many of these genes are involved in typical vertebrate pigmentation pathways, which we highlight in Fig. 5. In our analyses of gene function using all differentially expressed genes in PANTHER, we found that most of these genes were associated with either metabolic or cellular processes (Fig. 6). Similarly, most of these genes contributed to either cell part or organelle cellular components (Fig. 7). The molecular function was heavily skewed towards catalytic activity and binding, both of which are likely a result of the huge developmental reorganization involved in metamorphosis (Fig. 8).

**Table 2:** Differentially expressed candidate color genes in our transcriptome. Parentheses in the gene symbol column indicate the number of transcripts that mapped to a particular gene. The pathway column indicates what color or pattern production pathway this gene is a part of

Gene symbol	q value	Pathway	Citation
adam17 (2)	0.0163; 0.0469	Melanocyte development	[20]
arfgap1 (2)	0.00362; 0.0267	Putative guanine synthesis in iridophores	[26]
arfgap3 (4)	0.00739; 0.0000123; 0.00132; 0.0282	Putative guanine synthesis in iridophores	[26]
airc	0.0126	Guanine synthesis	[27]
atic	0.0447	Guanine synthesis in iridophores	[26]
atox1	0.00124	Melanogenesis	[28]
atp12a	0.0296	Melanogenesis	[29]
bbs2	0.0300	Melanosome transport	[30]
bbs5	0.0447	Melanosome transport	[30]
bmpr1b	0.0118	Inhibits melanogenesis	[31]
brca1	0.0455	Alters pigmentation, produces piebald appearances in mice	[32, 33]
ctr9	0.0280	Melanocyte assembly	[34, 35]
dera		Guanine synthesis in iridophores	[26]
dio2 (3)	0.0338; 0.0256; 0.000866	Thyroid hormone pathways, tenuous	[36]
dtnbp1 (2)	0.00120; 0.0456	Melanosome biogenesis	[37]
ednrb (2)	0.0035; 0.0005	Guanine synthesis in iridophores, melanoblast migration	[26, 38]
egfr (2)	0.0197; 0.000566	Melanocyte pigmentation and differentiation	[39]
fbxw4 (2)	0.00268; 0.0183	Melanophore organization	[40, 41]
gart	0.0000494	Purine synthesis, affecting iridophores, xanthophores, and melanophores	[42]
gas1 (2)	0.0264; 0.0191	Guanine synthesis in iridophores	[26]
gne (2)	0.00571; 0.0361	Sialic acid pathway	[43]
hps3	0.0202	Melanosome biogenesis	[18]
itgb1 (2)	0.0191; 0.0469	Guanine synthesis in iridophores	[26]
lef1	0.0190	Melanocyte differentiation and development, melanogenesis	[44]
leo1	0.0000381	Melanocyte assembly	[45]
mitf	0.0466	Melanocyte regulation	[46]
mlph	0.00568	Melanosome transport	[47]
mthfd1	0.0430	Purine synthesis	[48]
mreg	0.0156	Melanosome transport	[49]
notch1 (3)	0.00681; 0.0139; 0.0487	Melanocyte production	[50, 51]
prtfdc1	0.00000672	Guanine synthesis	[26]
qdpr	0.0372	Guanine and Pteridine synthesis	[52, 53]
qnr-71 (2)	0.0316; 0.0262	Melanosomal protein	[54, 55]
rab3d	0.0321	Putative guanine synthesis in iridophores	[26]
rab7a	0.0319	Putative guanine synthesis in iridophores	[26]
rabggta	0.000864	Guanine synthesis	[56]
scarb2	0.0329	Putative guanine synthesis in iridophores	[26]
shroom2	0.0142	Pigment accumulation	[57, 58]
sox9	0.0228	Melanin production	[59]
tbx15	0.00838	Pigmentation boundaries	[60]
tyrp1	0.0200	Melanogenesis	[61]
xdh (2)	0.0346; 0.0384	Pteridine synthesis	[62]

## Discussion

The genetic mechanisms of color variation are poorly known, particularly in amphibians. Here, we address this deficiency by providing some of the first genomic data relevant to color production in amphibians, with a focus on gene expression in the skin during development. Our model system and strategy support the identification of genes likely to regulate color and pattern elements across different morphs of a highly variable species. By combining analyses of differential expression with a targeted search based on an extensive list of candidate genes for developmental control of coloration (approximately 500 genes), we identified multiple genes that were differentially expressed among morphs which have been demonstrated to play important roles in the production of color in other taxa.

We found differential expression of multiple genes in two major suites of color genes, those that influence melanic coloration (black, brown, and grey) and iridophore genes (blue and green coloration). Additionally, we found a few key pteridine pigment genes that are known to influence primarily yellow amphibian coloration that were differentially expressed between morphs. Given that our color morphs had a black versus brown color coupled with either blue or green pattern elements on top of the background, these results seem biologically relevant and indicative of genes that control color and pattern in *Dendrobates auratus.* As a result, we divide our discussion into three main parts, focusing on the genes that influence dark background coloration, purine synthesis, and iridophore biology. We then discuss a few genes that are part of other pathways (e.g. pteridine synthesis), before proposing genes that have not previously been implicated in the production of color but are plausible candidate genes.

### Melanin-related gene expression

Our study frogs have skin with either a black or brown background, both of which are forms of melanic coloration, which provides the basis for contrasting patterns in many vertebrates as well as non-vertebrate taxa [63]. Melanin is synthesized from tyrosine in vertebrates, via the action of a set of key enzymes (e.g., tyrosinase, tyrosinase-like protein 1 and 2). We identified a suite of differentially expressed genes that are involved in the production of melanophores and melanin in this study (Figs. 6 and 8), many of which have been tied to the production of relatively lighter phenotypes in previous studies. Intriguingly, our results parallel similar findings in *Oophaga histrionica*, a species of poison frog in which mutations in the *mc1r* gene affecting melanogenesis have produced a lighter, more brownish background in some populations [64]. In a pattern reminiscent of their results, we found that *mc1r* was only lowly expressed in one super blue frog, and that a variety of other genes linked to lighter phenotypes followed a similar pattern of expression.

For example, many of the differentially expressed color genes in our dataset are active contributors to the tyrosinase pathway (*tyrp1, mitf, sox9, lef1, mlph, leo1, adam17, egfr, ednrb*). This pathway is enzymatically regulated by tyrosinase as well as other enzymes and cofactors and is key to the production of melanin [65]. The *tyrp1* enzyme catalyzes several key steps in the melanogenesis pathway in melanosomes (and melanocytes), has been shown to affect coloration in a wide variety of vertebrates [65, 66], and is important for maintaining the integrity of the melanocytes [67]. In some mammals *tyrp1* has been shown to change the relative abundances of the pigments pheomelanin and eumelanin, thereby producing an overall lighter phenotype [68]. Our data mimic this pattern as *tryp1* is not expressed in the blue-black morph, and only expressed at low levels in some San Felix individuals. Comparing the photos of the four morphs (Fig. 1), it can readily be seen that blue and black morph has substantially darker (black) background coloration, compared to the other three, which all have a lighter, brownish background coloration. Pheomelanin has only been identified in the skin of one species of frog [69], and it is unclear whether pheomelanin is present outside of mammals and birds. Further, mutations in *tyrp1* change melanic phenotypes through different mechanisms in fish (and possibly other ectotherms) than in mammals [66, 70], and the mechanisms by which *tyrp1* one affects pigmentation in amphibians are still being elucidated.

The *mitf* (microphthalmia-associated transcription factor) locus codes for a transcription factor that plays a dominant role in melanogenesis, and has been called the “master regulator” of melanogenesis [71]. In our study, *mitf* expression was lowest in the microspot population, the population with the least melanic coloration, and most highly expressed in the blue-black morph (although it is worth noting that blue and green colors are also influenced by melanin to some degree). The *mitf* locus is, itself, targeted by a suite of transcriptional factors including two which were differentially expressed in our dataset: *sox9* and *lef1*. The *sox9* gene is upregulated during melanocyte differentiation, can promote melanocyte differentiation, and has been demonstrated to be an important melanocytic transcription factor [72]. Further, *sox9* is up-regulated in human skin after UVB exposure and has been demonstrated to increase pigmentation. *Sox9* was not expressed in the microspot morph and was only expressed (at a low level) in one San Felix individual. Another important transcription factor is the lymphoid enhancer-binding factor locus (*lef1*), which mediates *Wnt* signaling in the context of melanocyte differentiation and development, with important effects on melanogenesis [44]. Upregulation of this gene has been found to reduce synthesis of the darkest melanic pigment eumelanin, resulting in lighter coloration in mink and other vertebrates [44]. In our study, *lef1* showed very low expression in the blue and black morph, compared to the other three morphs, indicating that *lef1* is a likely contributor to the background dorsal coloration between color morphs in *Dendrobates auratus*.

Just as *mitf* is a target of the transcription factors *lef1* and *sox9, mitf* targets endothelin receptors, a type of G Protein Coupled Receptor. Endothelin receptors mediate several crucial developmental processes, particularly the development of neural crest cell populations [73]. Three paralogous families of these receptors have been identified in vertebrates: endothelin receptor B1 (*ednrb1*), endothelin receptor B2 (*ednrb2*), and endothelin receptor A (*ednra*). *Ednrb* is involved in producing the different male color morphs of the Ruff (a sandpiper), and it is only expressed in black males [17]. In our study, *ednrb* is not expressed in the blue-black morph, and only one of the *ednrb* transcripts is expressed in the San Felix morph. Mutations in *ednrb1* and *ednrb2* have been found to affect pigment cell development (especially melanocytes and iridophores) in a variety of vertebrate species [73]. These receptors show divergent patterns of evolution in the ligand-binding region in African lake cichlids, and appear to have evolved divergently in association with adaptive radiations in this group [15]. The *ednrb2* (endothelin receptor B2) locus encodes a transmembrane receptor that plays a key role in melanoblast (a precursor cell of the melanocyte) migration [38]. This receptor interacts with the *edn3* ligand. Mutations affecting this ligand/receptor system in *Xenopus* affect pigment cell development [74].

The *leo1* (LEO1 Homolog) and *ctr9* (CTR9 Homolog) loci are both components of the yeast polymerase-associated factor 1 (*Paf1*) complex, which affects the development of the heart, ears and neural crest cells in zebrafish, with dramatic downstream effects on pigment cells and pigmentation, as well as on the Notch signaling pathway [34, 35]. Perhaps unsurprisingly then, we found that *notch1*, a well-known member of the Notch Signaling Pathway, was differentially expressed between color morphs. Mutations in this gene are known to affect skin, hair and eye pigmentation in humans through effects on melanocyte stem cells [50]. This indicates that *notch1* is a good candidate gene for pattern development in poison frogs.

A number of other melanogenesis-related genes were found to be differentially expressed between morphs, such as *brca1*. Mice with a homozygous mutation of the tumor suppressing *brca1* gene show altered coat coloration, often producing a piebald appearance [32]. The precise mechanism behind this is ambiguous, and it may involve either *mitf* or *p53* [33, 75]. *Bmpr1b* is a bone morphogenic protein which is known to inhibit melanogenesis; when *bmpr1b* is downregulated via UV exposure it enhances melanin production and leads to darker pigmentation [31]. Some of the other genes (e.g. *mlph,* or melanophilin) show the same pattern of expression across morphs as *lef1*, suggesting that multiple genes may contribute to the difference between lighter and darker background coloration in this species. The product of the melanophilin gene forms a complex that combines with two other proteins and binds melanosomes to the cell cytoskeleton, facilitating melanosome transport within the cell. Variants of this gene are associated with “diluted”, or lighter-colored, melanism in a number of vertebrates [47]. Similarly, the *mreg* (melanoregulin) gene product functions in melanosome transport and hence is intimately involved in pigmentation [49]. Mutations at this locus cause “dilute” pigmentation phenotypes in mice.

In summary, we have found a number of differentially expressed genes that influence melanic coloration which seem to be important between color morphs with a true, black background pattern versus those with a more dilute, brown colored background pattern. Our results parallel similar findings in *Oophaga histrionica*, a species of poison frog in which mutations in the *mc1r* gene affecting melanogenesis have produced a lighter, more brownish background in some populations [64]. In addition to *mc1r*, we have identified a suite of genes with the same expression pattern that are ultimately influenced by *mc1r* activity; many of these genes have been linked to lighter phenotypes in other taxa.

### Purine synthesis and iridophore genes

The bright coloration of *D. auratus* is confined to the green-blue part of the visual spectrum (with the exception of some brownish-white varieties) in most populations, and thus iridophores are likely to play a role in the color variation displayed across different populations of this species. Higdon et al. (2013) identified a variety of genes that are components of the guanine synthesis pathway and show enriched expression in zebrafish iridophores. A number of these genes (*hprt1, ak5, dera, ednrb2, gas1, ikpkg, atic, airc, prtfdc1*) were differentially expressed between the different morphs of *D. auratus* investigated here (Fig. 8). The *gart* gene codes for a tri-function enzyme that catalyzes three key steps in the de novo purine synthesis pathway [42]. This locus has been associated with critical mutations affecting all three types of chromatophores in zebrafish, through effects on the synthesis of guanine (iridophores), sepiapterin (xanthophores) and melanin (melanocytes) [42]. Zebrafish mutants at this locus can show dramatically reduced numbers of iridophores, resulting in a lighter, or less saturated color phenotype. Similarly, the *airc* gene plays a critical role in guanine synthesis, and yeast with mutations in this gene leading to aberrant forms of the transcribed protein are unable to synthesize adenine and accumulate a visible red pigment [27, 76]. Similarly, the *mthfd* (methylenetetrahydrofolate dehydrogenase, cyclohydrolase and formyltetrahydrofolate synthetase 1) gene also affects the de novo purine synthesis pathway [77]. The genes *airc, gart,* and *mthfd* had similar expression patterns and were very lowly expressed in the mostly green microspot population. The gene *prtfdc1* is highly expressed in iridophores, and encodes an enzyme which catalyzes the final step of guanine synthesis [26]; *prtfdc1* had very low expression in the dark blue-black morph, which may be an indication that it plays a role in the reflectance from iridophores. Further, *prtfdc1* was highly expressed in the San Felix and super blue morphs, both of which have visible small white ‘sparkles’ on the skin which are likely produced by the iridophores.

How the guanine platelets are formed in iridophores remains an open question. Higdon et al. (2013) proposed that ADP Ribosylation Factors (ARFs) and Rab GTPases are likely to play crucial roles in this context. ARFs are a family of ras-related GTPases that control transport through membranes and organelle structure. We identified one ARF protein (*arf6*) and two ARF activating proteins (*arfgap1* and *arfgap2*) that were differentially expressed across the *D. auratus* morphs. We also identified four different Rab GTPases as differentially expressed (*rab1a, rab3c, rab3d, rab7a*). Mutations at the *rabggta* (Rab geranylgeranyl transferase, a subunit) locus cause abnormal pigment phenotypes in mice (e.g. “gunmetal”), are known to affect the guanine synthesis pathway [18], and are similarly differentially expressed between color morphs in our dataset. These genes are likely candidates to affect coloration in *Dendrobates auratus* given that both the green and blue pattern elements are probably iridophore-dependent colors.

### Pteridine synthesis

Above we have devoted a large amount of space to melanophore and iridophore related genes. Here we will briefly discuss pteridine synthesis genes, because there is generally less known about them and there are fewer pteridine genes differentially expressed between color morphs in our study. A number of the genes identified as differentially expressed are involved in copper metabolism (*sdhaf2*, *atox1*, *atp7b*). Copper serves as a key cofactor for tyrosinase in the melanogenesis pathway and defects in copper transport profoundly affect pigmentation [28]. Another gene, the xanthine hydrogenase (*xdh*) locus, was also found to be differentially expressed between morphs, and this gene, which is involved in the oxidative metabolism of purines, affects both the guanine and pteridine synthesis pathways. Additionally, it has been shown to be critically important in the production of color morphs in the axolotl. When *xdh* was experimentally inhibited axolotls had reduced quantities of a number of pterins, and also showed dramatic differences in color phenotype with *xdh*-inhibited individuals showing a ‘melanoid’ (black) appearance [62]. Furthermore, *xdh* deficient frogs show a blue coloration in a species that is typically green [78, 79]. We note here that one *xdh* transcript showed little (one individual) or no (2 individuals) expression in the bluest morph (blue-black). Similarly, when pigments contained in the xanthophores that absorb blue light are removed, this can lead to blue skin [23]. We also found another gene involved in pteridine synthesis, *qdpr* (quinoid dihydropteridine reductase), was only expressed in the populations with a lighter blue or green coloration. Mutations in this gene result in altered patterns of pteridine (e.g. sepiapterin) accumulation [53]. We believe that *xdh* and *qdpr* are good candidates for variability in coloration in poison frogs.

### Fixed genomic variants

Similar to our analysis of differentially expressed color genes, we found a number of SNPs in melanophore and iridophore related genes with alternate fixation among color morphs. For example, the cappuccino gene (*cno*) is known to effect the maturity of melanosomes and can also dramatically influence the size and number of melanosomes, which produces dramatic changes in phenotypes and can lead to albinism [80]. Intriguingly, *cno* alleles are alternately fixed between the microspot and San Felix populations, the latter of which has an almost cappuccino-colored background coloration. Similarly, type II iodothyronine deiodinase (*dio2*) is involved in thyroid hormone conversion, and in flounders it is thought that this conversion promotes pigmentation and prevents albinism [81, 82]. *Dio2* is also known to play a role in vision via the pigmentation of the retinal pigment epithelium [83, 84], as are a number of other genes with alternately fixed alleles (*rlbp1, ebna1bp2;* [85, 86]). Given the close link between eyesight and pigmentation generally, these genes could undergo similar coevolutionary paths in poison frog diversification. We also found fixed differences in *prtfdc1,* a gene which is responsible for the final step of guanine synthesis and is highly expressed in iridophores [26], and the fixed difference in this gene may be associated with darker versus lighter blue frogs. Another iridophore gene, *pgm2* (Phosphoglucomutase-2), had the highest overall number of fixed SNPs in our study (8 SNPs), all of which are fixed differences between the super blue morph and the blue-black/microspot morphs. This gene is highly expressed in iridophores when compared to melanin or retinal pigment epithelium cells [26], so these variants are plausible determinants of blue coloration in *Dendrobates auratus*. In addition to genes related to pigment production, we also saw fixed genomic variants of genes dealing specifically with patterning. For example, spermidine (*srm*) exhibited fixed differences between color morphs, and this gene is essential for pigment patterning in zebrafish [87]. Further, we saw fixed differences in two SNPs of the gene *rtf1*, a gene which is known to interact with the Notch signalling pathway and modulate pigmentation and striping in zebrafish [34]. We also found that *notch1* was differentially expressed between color morphs in our dataset. Thus, the combination of *srm* and *rtf1* SNPs and differential expression of *notch1* indicate that these genes may play a role in the divergence of pattern elements among color morphs. Most of the color morph specific SNPs we found in candidate color genes appear to produce non-synonymous changes in the amino acid sequence. In fact, every color gene with a fixed difference in SNPs had at least one non-synonymous change except for *pts* and *dio2,* the latter of which we were unable to find a matching amino acid sequence for. This provides further evidence that these morph-specific fixed variants are contributing to color and pattern differences in *Dendrobates auratus.* These fixed, non-synonymous changes also indicate that these genes may be under positive selection to be maintained within color morphs. However, the possibility remains that patterns of alternate fixation of alleles in our inter-population comparisons are due to genetic drift, or selection on alleles due to their impact on traits other than pigmentation.

### Novel candidate genes for coloration

In addition to those genes that have previously been linked to coloration which we have identified in our study, we would like to propose several others as candidate color genes, based on their expression patterns in our data. Although most research on blue coloration focuses on light reflecting from iridophores, this has generally not been explicitly tested and there is some evidence that blue colors may arise through different mechanisms (reviewed in [23]). In particular, there is evidence that blue in amphibians can come from the collagen matrix in the skin, as grafts in which chromatophores failed to thrive show a blue coloration [23]. Furthermore, keratinocytes surround melanocytes, and they play a key role in melanosome transfer [88]. In light of this evidence, we propose a number of keratinocyte and collagen genes which are differentially expressed in our dataset as further candidate genes for coloration. Amongst these are *krt12,* and *krt8, col1a1*, *col5a1*, and *col14a1*. Indeed, alleles of one of these genes, *krt8,* are differentially fixed between color morphs. These genes, and those like them, may be playing a critical role in coloration in these frogs.

## Conclusion

The mechanisms that produce variation in coloration in both amphibians and aposematic species are poorly characterized, particularly in an evolutionary context. Here we have taken a transcriptomics-based approach to elucidating the genetic mechanisms underlying color and pattern development in a poison frog. We found evidence that genes characterizing the melanin and iridophore pathways are likely the primary contributors to color and pattern differences in this aposematic species. Additionally, a handful of genes which contribute to the pteridine pathway seem to be playing a role in differential color production as well. However, the specific mechanisms by which these genes work, as well as how they interact to produce color phenotypes, remains an outstanding issue given the complex nature of each of these pathways. Still, our data indicate that genes involved at every step along the melanin and iridophore pathways from chromatophore production, through pigmentation production and deposition, influence differences in coloration between these morphs. These results make sense in the context of the overall color and pattern of these frogs, and provide a number of promising starting points for future investigations of the molecular, cellular and physiological mechanisms underlying coloration in amphibians.

## Methods

### Color morphs

Captive bred *Dendrobates auratus* were obtained from Understory Enterprises, LLC. We note that the breeding stock of these different morphs, while originally derived from different populations in Central America, have been bred in captivity for many generations. As a result, it is possible that color pattern differences between these morphs in captivity may exceed those generally found in the original populations. Nevertheless, the differences between these morphs are well within the range of variation in this highly variable, polytypic species which ranges from Eastern Panama to Nicaragua.

### Sample collection

Frogs were maintained in pairs in 10 gal tanks with coconut shell hides and petri dishes were placed under the coconut hides to provide a location for females to oviposit. Egg clutches were pulled just prior to hatching and tadpoles were raised individually in ~ 100 mL of water. Tadpoles were fed fish flakes three times a week, and their water was changed twice a week. Froglets were sacrificed during the final stages of aquatic life (Gosner stages 41–43; [89]). At this point, froglets had both hind limbs and at least one forelimb exposed. These froglets had color and pattern elements at this time, but pattern differentiation and color production is still actively occurring during metamorphosis and afterwards. Individuals were anesthetized with 20% benzocaine gel applied to the venter, followed by double pithing to ensure death. After euthanasia, whole specimens (*n* = 3 per morph) were placed in RNAlater (Qiagen) for 24 h, prior to storage in liquid nitrogen. We then did a dorsal bisection of each frog’s skin, and prepared half of the skin for RNA extraction.

RNA was extracted from each bisected dorsal skin sample using a hybrid Trizol (Ambion) and RNeasy spin column (Qiagen) method and total RNA quality was assayed using the Bioanalyzer 2100 (Agilent). Messenger RNA (mRNA) was isolated from total RNA with Dynabeads Oligo(dT)_25_ (Ambion) for use in the preparation of uniquely-barcoded, strand-specific directional sequencing libraries with a 500 bp insert size (NEBNext Ultra Directional RNA Library Prep Kit for Illumina, New England Biosystems). Libraries were placed into a single multiplexed pool for 300 bp, paired end sequencing on the Illumina MiSeq. Each sample had a total of 2–5.8 million reads, as a result sequencing depth is a limiting factor in our analyses.

### Transcriptome assembly

We randomly chose one individual per morph type and assembled this individual’s transcriptome. First, we aggressively removed adaptors and did a gentle quality trimming using trimmomatic version 0.36 [90]. We then implemented read error correction using RCorrector version 1.01 [91] and assembled the transcriptome using the Oyster River Protocol version 1.1.1 [25]. Transcriptomes were assembled using Trinity version 2.4.0 [92], two independent runs of SPAdes assembler version 3.11 with kmer lengths of 55 and 75 [93], and lastly Shannon version 0.0.2 with a kmer length of 75 [94]. The four transcriptomes were then merged together using OrthoFuser [25]. Transcriptome quality was assessed using BUSCO version 3.0.1 against the eukaryote database [95] and TransRate 1.0.3 [96]. BUSCO evaluates the genic content of the assembly by comparing the transcriptome to a database of highly conserved genes. Transrate contig scores evaluate the structural integrity of the assembly, and provide measures of accurate, completeness, and redundancy. We then compared the assembled, merged transcriptome to the full dataset (every read in our dataset concatenated together) by using BUSCO and TransRate. We recognize that the data used for transcriptome assembly greatly influences downstream analyses, especially in experimental work in which certain genes may only be expressed in one treatment. However, we did limit the likelihood of this by choosing one individual per color morph. Evidence indicates that our approach did successfully address this issue, as our transcriptome has a very high BUSCO score (> 95%).

### Downstream analyses

We annotated our transcriptome using the peptide databases corresponding to frog genomes for *Xenopus tropicalis* [97], *Nanorana parkeri* [98], and *Rana catesbeiana* [99] as well as the UniRef90 database [100] using Diamond version 0.9.10 [101] and an e-value cutoff of 0.001. We then pseudo-aligned reads from each sample using Kallisto version 0.43.0 [102] and examined differential expression of transcripts in R version 3.4.2 [103] using Sleuth version 0.29.0 [104]. Differential expression was analyzed by performing a likelihood ratio test comparing a model with color morph as a factor to a simplified, null model of the overall data, essentially testing for differences in expression patterns between any of the four morphs. In addition to examining overall differential expression between morphs, we examined differential expression in an a priori group of candidate color genes. We used PANTHER [105] to quantify the distribution of differentially expressed genes annotated to *Xenopus tropicalis* into biological processes, molecular functions, and cellular components. Finally, we used ANGSD for an analysis of SNPs [106]. We only examined SNPs that had a minimum quality score of 20, and a minimum depth of 100 reads. Following SNP calling, we examined SNPS that were fixed in at least one color morph and were in our candidate color gene list. We then used BLAST translated nucleotide to protein searches (tblastx) to align the color morph specific gene variants to the best amino acid sequence match in the model species genome (either *Xenopus* or *Nanorana*). We confirmed codon frame by aligning the specific protein sequence from the model species (*Xenopus* or *Nanorana*) to the matching translated nucleotide sequence for each candidate gene in *D. auratus* (except in the case of *dio2,* for which we were unable to find a matching amino acid sequence). We then determined whether the color morph specific fixed variants produced synonymous or non-synonymous changes or introduced stop codons.

## Additional files

Additional file 1: **Table S1.** Differentially expressed genes. A list of genes that were differentially expressed and relevant test statistics. (CSV 482 kb)

Additional file 2: **Table S2.** Fixed genomic variants of color genes. Contig, SNP position, gene name, and whether these changes produce synonymous or non-synonymous changes across color morphs. (CSV 2 kb)

## Abbreviations

adam17: A disintegrin and metalloprotease domain 17
airc: Phosphoribosylaminoimidazole Carboxylase And Phosphoribosylaminoimidazolesuccinocarboxamide Synthase
arfgap1: aDP Ribosylation Factor GTPase Activating Protein 1
arfgap3: aDP Ribosylation Factor GTPase Activating Protein 3
atic: 5-Aminoimidazole-4-Carboxamide Ribonucleotide Formyltransferase/IMP Cyclohydrolase
atox1: Antioxidant 1 Copper Chaperone
atp12a: aTPase H+/K+ Transporting Non-Gastric Alpha2 Subunit
bbs2: Bardet-Biedl Syndrome 2
bbs5: Bardet-Biedl Syndrome 5
bmpr1b: Bone morphogenic protein 1
brca1: Breast And Ovarian Cancer Susceptibility Protein 1
cno: Cappuccino
col14a1: Collagen Type XIV Alpha 1 Chain
col1a1: Collagen Type I Alpha 1 Chain
col5a1: Collagen Type V Alpha 1 Chain
ctr9: cTR9 Homolog
dera: Deoxyribose-Phosphate Aldolase
dio2: Type II iodothyronine deiodinase
dtnbp1: Dystrobrevin Binding Protein 1
edn3: Endothelin 3
ednrb: Endothelin receptor B2
egfr: Epidermal Growth Factor Receptor
fbxw4: f-Box And WD Repeat Domain Containing 4
gart: Phosphoribosylglycinamide Formyltransferase
gas1: Growth Arrest Specific 1
gne: Glucosamine (UDP-N-Acetyl)-2-Epimerase/N-Acetylmannosamine Kinase
hps3: Hermansky-Pudlak Syndrome 3 Protein
itgb1: Integrin Subunit Beta 1
krt12: Keratin 12
krt8: Keratin 8
lef1: Lymphoid Enhancer Binding Factor 1
leo1: lEO1 Homolog
mc1r: Melanocortin 1 Receptor
mitf: Microphthalmia-associated transcription factor
mlph: Melanophilin
mreg: Melanoregulin
mthfd1: Methylenetetrahydrofolate dehydrogenase
notch1: Neurogenic locus notch homolog protein 1
paf1: Yeast polymerase-associated factor 1
pgm2: Phosphoglucomutase-2
prtfdc1: Phosphoribosyl Transferase Domain Containing 1
pts: 6-Pyruvoyltetrahydropterin Synthase
qdpr: Quinoid dihydropteridine reductase
qnr-71: quail Neuroretina clone 71
rab3d: Rab3d, Member RAS Oncogene Family
rab7a: Rab7a, Member RAS Oncogene Family
rabggta: Rab geranylgeranyl transferase, a subunit
scarb2: Scavenger Receptor Class B Member 2
shroom2: Shroom Family Member 2
SNP: Single nucleotide polymorphism
sox9: Sex determining region box 9
srm: Spermidine
tbx15: t-Box 15
tyrp1: Tyrosinase Related Protein 1
Wnt: Wingless/Integrated
xdh: Xanthine hydrogenase
